# Genetic research and clinical analysis of deletional Chinese ^G^γ^+^(^A^γδβ)^0^ -thalassemia and Southeast Asian HPFH in South China

**DOI:** 10.1007/s00277-020-04252-7

**Published:** 2020-09-15

**Authors:** Yuanjun Wu, Qianyu Yao, Ming Zhong, Jianying Wu, Longxu Xie, Linnan Su, Fubing Yu

**Affiliations:** 1 Department of Pre-marital and Pre-pregnancy Eugenic Health Examination Center, Dongguan Maternal and Child Health Hospital, Guangdong, China; 2 Department of Blood Transfusion, Dongguan Maternal and Child Health Hospital, Guangdong, China; 3 Guangdong Hybribio Biotech Co., Ltd, Guangdong, China; 4 Department of Obstetrics and Gynecology, Dongguan Maternal and Child Health Hospital, Guangdong, China

**Keywords:** Chinese ^G^γ^+^(^A^γδβ)^0^-thalassemia, SEA-HPFH, Genetic diagnosis

## Abstract

Chinese ^G^γ^+^(^A^γδβ)^0^-thalassemia and SEA-HPFH are the most common types of β-globin gene cluster deletion in Chinese population. The aim of the study was to analyze clinical features of deletional Chinese ^G^γ^+^(^A^γδβ)^0^-thalassemia and Southeast Asian hereditary persistence of fetal hemoglobin (SEA-HPFH) in South China. A total of 930 subjects with fetal hemoglobin (HbF) level ≥ 2% were selected on genetic research of Chinese ^G^γ^+^(^A^γδβ)^0^-thalassemia and SEA-HPFH. The gap polymerase chain reaction was performed to identify the deletions. One hundred cases of Chinese ^G^γ^+^(^A^γδβ)^0^-thalassemia were detected, including 90 cases of Chinese ^G^γ^+^(^A^γδβ)^0^/β^N^-thalassemia, 7 cases of Chinese ^G^γ^+^(^A^γδβ)^0^ /β^N^-thalassemia combined with α-thalassemia, 2 cases of Chinese ^G^γ^+^(^A^γδβ)^0^-thalassemia combined with β-thalassemia, and 1 case of Chinese ^G^γ^+^(^A^γδβ)^0^-thalassemia combined with β-gene mutation. One hundred nine cases of SEA-HPFH were detected, including 97 cases of SEA-HPFH/β^N^, 9 cases of SEA-HPFH/β^N^ combined with α-thalassemia, 2 cases of SEA-HPFH combined with β-thalassemia, and 1 case of SEA-HPFH combined with β-gene mutation. Statistical analysis indicates significant differences in MCV (mean corpuscular volume), MCH (mean corpuscular hemoglobin), and HbA2 and HbF levels between Chinese ^G^γ^+^(^A^γδβ)^0^-thalassemia heterozygotes and SEA-HPFH heterozygotes (*P* < 0.001). There are statistical differences in hematological parameters between them. Clinical phenotypic analysis can provide guidance for genetic counseling and prenatal diagnosis.

## Introduction

Hemoglobin is a special protein that transports oxygen within red blood cells, composed of two α and two β chains. Deviant hemoglobin can lead to two common diseases: abnormal hemoglobin and thalassemia. Thalassemia is one of the most common monogenic autosomal recessive genetic diseases [1, 2]. There are two main kinds of thalassemia: α- and β-thalassemias depending on the aberrant hemoglobin gene. Furthermore, α-thalassemias are mainly caused by the gene deletion, and β-thalassemias are primarily resulted from gene mutation. The mechanisms of both kinds of thalassemias are imbalance of hemoglobin chains and ineffective hematopoiesis [3]. Until now, we only have limited treatment options, of which transfusion and iron chelation are the main methods. However, they are mostly supportive [3]. Even the most promising effective therapy—hematopoietic stem cell transplantation—is only affordable for a small number of people [4]. Prenatal diagnosis and genetic counseling play a key role in the prevention of thalassemia [5].

The thalassemia has various frequencies and many kinds of gene types all over the world, and different countries and ethnic have their own hotspot mutation types. China is a multi-ethnic country with a vast population which accounts for one fifth of the world’s population. A recent study reported a novel α-thalassemia deletion -α6.9 in the Chinese population [6]. And beta thalassemia is mainly caused by point mutations of beta globin gene, and a small part is caused by the deletion of the beta globin gene cluster [7].

The most common beta globin gene cluster deletion are Chinese ^G^γ^+^(^A^γδβ)^0^-thalassemia and Southeast Asian hereditary persistence of fetal hemoglobin (SEA-HPFH) in the Chinese population [8]. The deletion length of SEA-HPFH is about 27 kb, and the range includes the β-gene of the β-globin gene cluster and its 3-HS-1 region [9]. The SEA-HPFH heterozygotes have no clinical symptoms, and hematological parameters are in the normal range or in borderline level. The Chinese ^G^γ^+^(^A^γδβ)^0^-thalassemia has a deletion range of about 78.9 kb, including some ^A^γ globin genes, all δ and β-globin genes and DNA sequences with regulatory functions far downstream of the β-globin gene[10]. However, few research focus on the clinical features on these two common types of thalassemia. Loss of the β-globin gene cluster or mutation of the γ gene regulatory region results in the continuous expression of the γ gene. Therefore, a significant increase in HbF levels in adults usually indicates the absence of the β-globin gene cluster.

At present, the research about HbF increase has been reported widely. It is found that gene mutations and deletions, which may lead to the increase of HbF, have a varying effect on different races [11–13]. The aim of this study was to analysis the frequency of Chinese ^G^γ^+^(^A^γδβ)^0^-thalassemia and SEA-HPFH in South China. In this study, we also performed the analysis of clinical features between the two common thalassemias. These findings are important for providing information for genetic counseling and prenatal diagnosis.

## Materials and methods

### Patients

Between January 1, 2017 and December 30, 2019, a total of 930 unrelated subjects with increased HbF levels (≥ 2%) from south of China were included in this study. Information sheets regarding nationality, gender, age, dialect, natives, and written consent forms were available in Chinese to ensure comprehensive understanding of the study objectives. Informed consents were signed or thumb printed by the participants. Hematological parameters were measured using the Sysmex XE-2100 automated blood cell counter (Sysmex, Kobe, Japan). Hb analysis was performed using an automated capillary electrophoresis system (Sebia, Paris, France).

### Genetic analysis

Genomic DNA were extracted from peripheral blood leukocytes using DNA blood extraction kits (Hybribio Biochemistry Co., Ltd., Chaozhou, China). Molecular study for common alpha and beta defects in Chinese population were performed as previously described [14]. The gap polymerase chain reaction (Gap-PCR) and sequence analysis were then performed to identify the deletions using the respective flanking primers (Table 1). Probes and reaction mixture for ligation and PCR were purchased from MRC-Holland (SALSA MLPA kit P102 HBB; MRC-Holland, Amsterdam, the Netherlands).

**Table 1:** Primer sequences

	Primer	Primer sequence	Length
^G^γ^+^(^A^γδβ)^0^	F	GGCATATATTGGCTCAGTCA	20
	R	TCAACAATTATCAACATTACACC	23
SEA-HPFH	F	TGGTATCTGCAGCAGTTGCC	20
	R	AGCCTCATGGTAGCAGAATC	20

### Statistical analysis

Statistical analysis was conducted using the SPSS software (Ver. 13, SPSS Inc., Chicago, USA). Groups were compared using *t* test or analysis of variance.

## Results

### Hematological index analysis and genetic diagnosis results of Chinese ^G^γ^+^(^A^γδβ)^0^-thalassemia

One hundred cases of Chinese ^G^γ^+^(^A^γδβ)^0^-thalassemia were detected, including 90 cases of Chinese ^G^γ^+^(^A^γδβ)^0^/β^N^ -thalassemia, 7 cases of Chinese ^G^γ^+^(^A^γδβ)^0^ /β^N^-thalassemia combined with α-thalassemia, 2 cases of Chinese ^G^γ^+^(^A^γδβ)^0^-thalassemia combined with β-thalassemia, and 1 case of Chinese ^G^γ^+^(^A^γδβ)^0^-thalassemia combined with β-gene mutation. The blood cell analysis showed that the MCV, MCH, and HbA2 levels were decreased in Chinese ^G^γ^+^(^A^γδβ)^0^ /β^N^ cases, and the expression of HbF was significantly increased, and the average HbF level was 14.95%. Hematological indicators analysis and genetic diagnosis results of Chinese ^G^γ^+^(^A^γδβ)^0^-thalassemia are shown in Table 2.

**Table 2:** Hematological and gene diagnosis data of 100 individuals with Chinese ^G^γ^+^(^A^γδβ)^0^-thalassemia

Genotype (a-thalassemia)	Genotype (β-thalassemia)	*n*	MCV (fL)	MCH (pg)	HbA2 (%)	HbF (%)
αα/αα	^G^γ^+^(^A^γδβ)^0^	90	71.41 ± 4.7	23.1 ± 1.93	2.47 ± 0.23	14.95 ± 3.30
-α^3.7^/αα	^G^γ^+^(^A^γδβ)^0^	3	69.35 ± 3.32	23.4 ± 1.41	2.45 ± 0.07	14.3 ± 4.24
--^SEA^/αα	^G^γ^+^(^A^γδβ)^0^	2	74.1 ± 2.77	23.2 ± 0.34	2.41 ± 0.15	9.35 ± 1.80
α^ws^α/αα	^G^γ^+^(^A^γδβ)^0^	1	78.3	24.1	2.23	17.88
αα/αα	^G^γ^+^(^A^γδβ)^0^/β^CapM^	1	76.4	24.4	2.5	15.9
-α^3.7^/αα(HBA2:c.*71G > C)	^G^γ^+^(^A^γδβ)^0^	1	68.6	20.6	2.5	12.1
αα/αα	^G^γ^+^(^A^γδβ)^0^/β^CD113^	1	68.9	22.6	2.09	21.89
αα/αα	^G^γ^+^(^A^γδβ)^0^/β^17^	1	/	/	1.76	74.63

### Hematological index analysis and genetic diagnosis results of SEA-HPFH

**Fig. 1:**
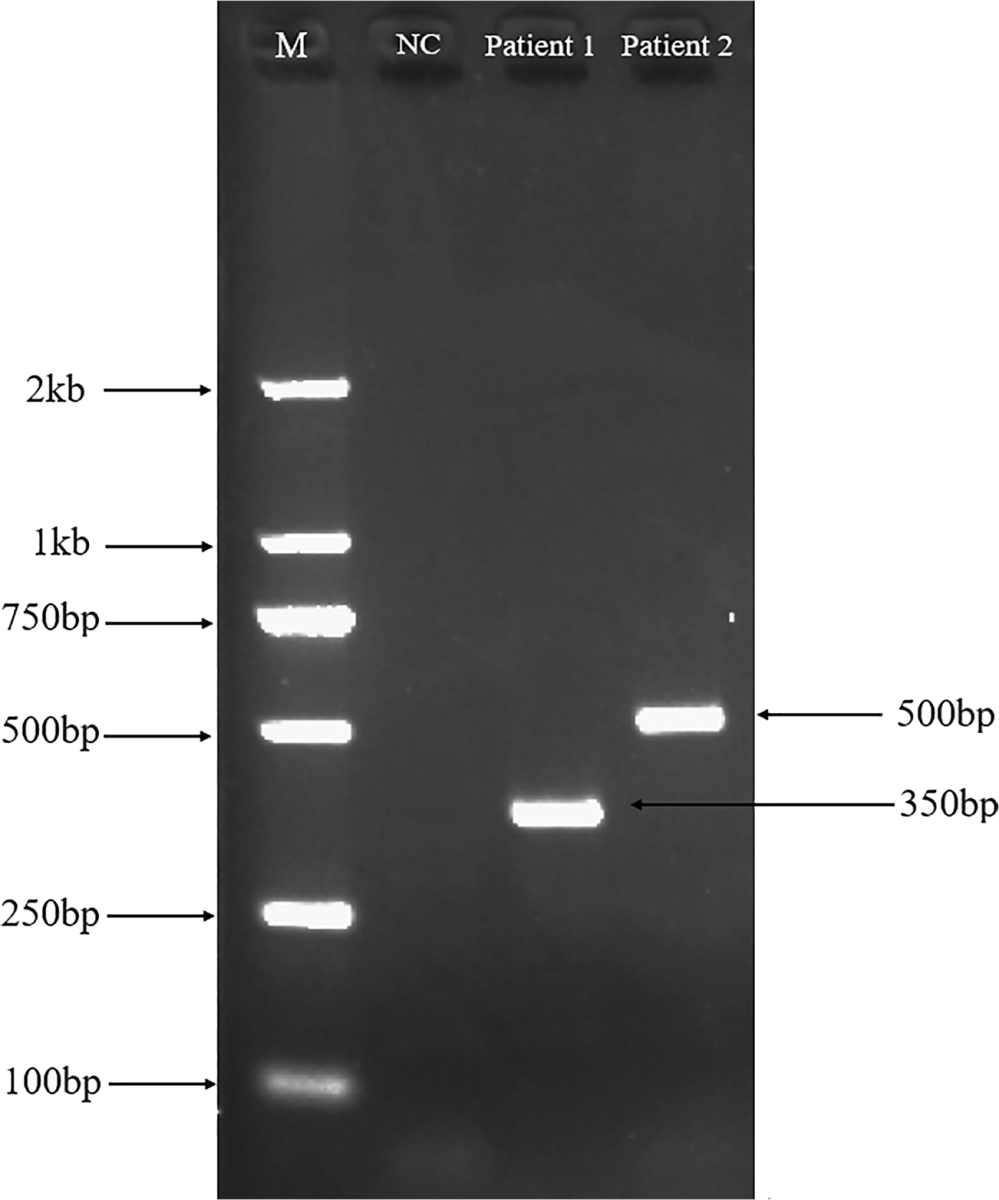
The representative map of Gap-PCR electrophoresis of patient 1 (SEA-HPFH) and patient 2 (^G^γ^+^(^A^γδβ)^0^-thalassemia)

**Fig. 2:**
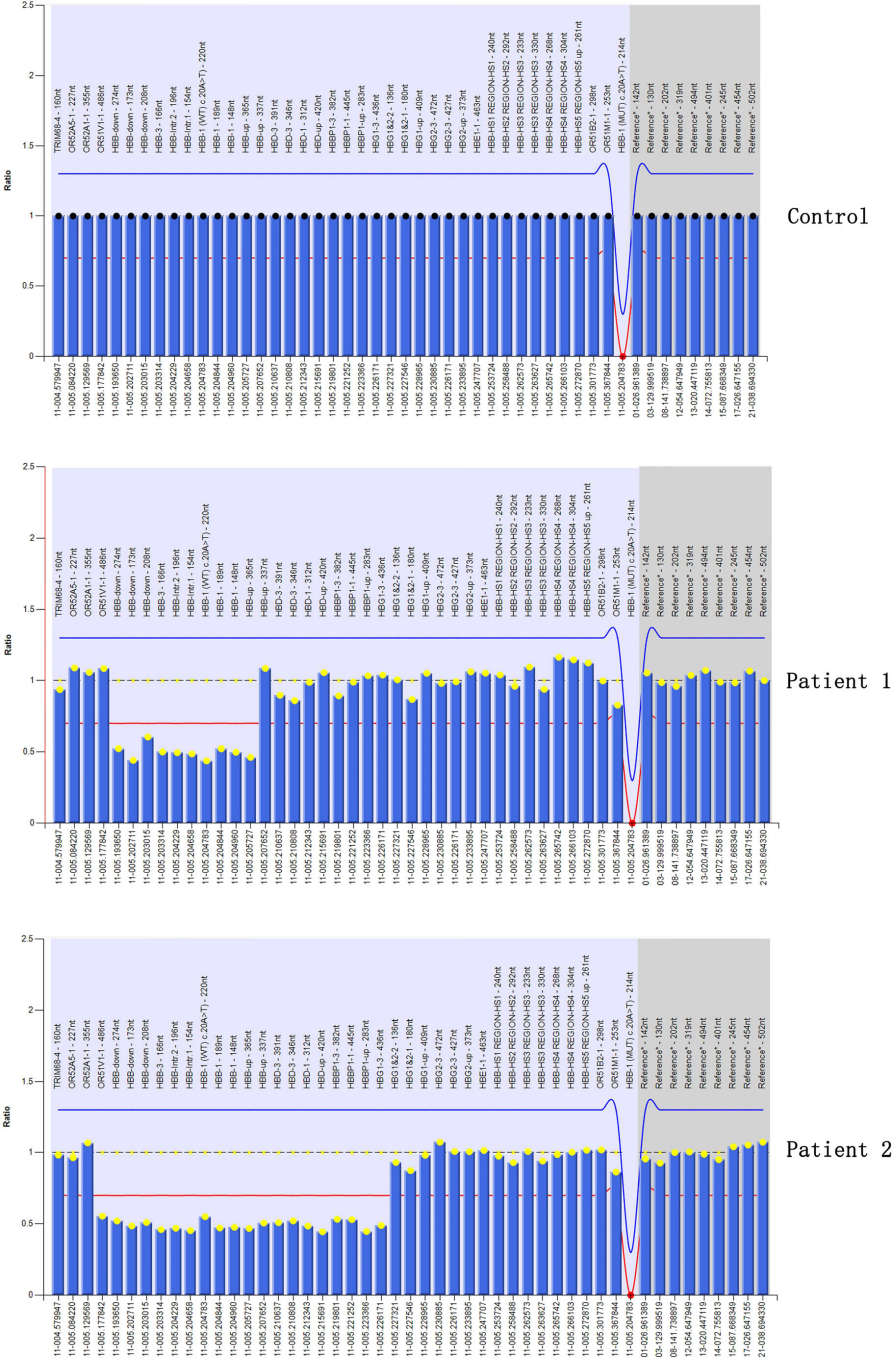
MLPA analysis showed half dosages for ten probes located in the HBB in patient 1 (SEA-HPFH) and twenty probes located in the HBB, HBD, and HBBP1 in patient 2 (^G^γ^+^(^A^γδβ)^0^-thalassemia)

One hundred nine cases of SEA-HPFH were detected, including 97 cases of SEA-HPFH/β^N^, 9 cases of SEA-HPFH/β^N^ combined with α-thalassemia, 2 cases of SEA-HPFH combined with β-thalassemia, and 1 case of SEA-HPFH combined with β-gene mutation. The blood cell analysis showed that the MCV of patients with SEA-HPFH/β^N^ was slightly decreased or normal, the level of HbA2 was increased, the level of HbF was significantly increased, and the average HbF content was 21.53%. Hematological indicator analysis and genetic diagnosis results of SEA-HPFH are shown in Table 3. The representative data of Gap-PCR electrophoresis and MLPA analysis are shown in Figs. 1 and 2, respectively.

**Table 3:** Hematological and gene diagnosis data of 109 individuals with SEA-HPFH

Genotype (a-thalassemia)	Genotype (β-thalassemia)	n	MCV (fL)	MCH (pg)	HbA2 (%)	HbF (%)
αα/αα	SEA-HPFH	97	76.6 ± 5.86	25.2 ± 1.93	3.9 ± 1.00	21.53 ± 6.3
--SEA/αα	SEA-HPFH	7	72.4 ± 4.39	23.29 ± 1.09	3.98 ± 0.93	15.55 ± 6.95
-α3.7/αα	SEA-HPFH	1	68.50	27.90	4.70	15.00
-α2.4/αα	SEA-HPFH	1	72.50	23.50	4.00	26.50
αα/αα	SEA-HPFH/β^41-42^	1	73.30	21.10	3.20	/
αα/αα	SEA-HPFH/β^(IVS II-180 T > C)^	1	75.40	24.40	/	/
αα/αα	SEA-HPFH/β^-43(C > T)^	1	79.00	24.90	2.30	15.10

### Comparison of hematological parameters between Chinese ^G^γ^+^(^A^γδβ)^0^/β^N^-thalassemia carriers and SEA-HPFH/β^N^ carriers

A total of 90 Chinese ^G^γ^+^(^A^γδβ)^0^/β^N^-thalassemia carriers and 97 SEA-HPFH/β^N^ carriers were analyzed statistically. The results showed that their differences in MCV, MCH, HbA2, and HbF levels were statistically significant. The data is shown in Table 4. MCV < 80 or MCH < 27 were used as cut-off values for thalassemia screening, and the normal reference intervals for HbA2 and HbF were 2.5–3.5% and less than 1.0%, respectively, in several studies based on Chinese population [15, 16].

**Table 4:** Results of hematologic analyses of 90 carriers with Chinese ^G^γ^+^(^A^γδβ)^0^-thalassemia and 97 carriers with SEA-HPFH

Index	Chinese ^G^γ^+^(^A^γδβ)^0^/β^N^	SEA-HPFH/β^N^	*P*
MCV (fl)	71.41 ± 4.7	76.6 ± 5.86	< 0.0001
MCH (pg)	23.1 ± 1.93	25.2 ± 1.93	< 0.0001
HbA2 (%)	2.47 ± 0.23	3.9 ± 1.00	< 0.0001
HbF (%)	14.95 ± 3.30	21.53 ± 6.3	< 0.0001

## Discussions

Two hundred different thalassemia mutations causing β-thalassemia have been reported in various ethnic groups and geographical regions (http://globin.cse.psu.edu/), including single nucleotide substitutions, deletions, or insertions of a few nucleotides leading to frameshift mutations [17]. At present, commercial kits in China usually only can detect the 17 most common point mutations of β-thalassemia and do not include Chinese ^G^γ^+^(^A^γδβ)^0^-thalassemia and SEA-HPFH, which are common in Chinese. So there is a possibility that such deletional β-thalassemia can be missed in routine genetic testing. For example, SEA-HPFH may coexist with point mutation of α- or β-thalassemia [18]. Moreover, compound heterozygotes of such deletional mutations with beta-thalassemia point mutations can result in intermediate or severe beta thalassemia. Therefore, it is necessary to identify the two types of deletion and analyze the corresponding clinical features. It is worth mentioning that clinical progress of gene therapy has made it possible to overcome β-thalassemia [19]. The results of a clinical trial of luspatercept for thalassemia in Italy showed that hemoglobin levels of several treated patients reached or approached normal ranges [20].

In this study, we analyzed the clinical phenotypic of Chinese ^G^γ^+^(^A^γδβ)^0^-thalassemia and Southeast Asian HPFH. The study results show that the levels of MCV and MCH of Chinese ^G^γ^+^(^A^γδβ)^0^ heterozygous are significantly reduced, which is a typical characteristic of mild thalassemia with small cell hypochromic anemia. The Chinese ^G^γ^+^(^A^γδβ)^0^ and SEA-HPFH deletion both have raised HbF level. The HbF level of heterozygous ^G^γ^+^(^A^γδβ)^0^ patients was about 14.95% and the heterozygous ^G^γ^+^(Aγδβ)^0^ ones was 21.53%; the parameters showed a significant difference. Moreover, when the heterozygous ^G^γ^+^(^A^γδβ)^0^-thalassemia coexist with β^17^, the HbF level can reach 74.63%. Although there was limited data to support this argument, it can provide some support and reference for clinical diagnosis. The HbA2 levels were normal or decreased in patients with Chinese ^G^γ^+^(^A^γδβ)^0^-thalassemia, while increased in heterozygous SEA-HPFH ones. And the difference was statistically significant. These clinical features provide valuable clues to the diagnosis. From the above results, the clinical phenotype of SEA-HPFH is less severe than that of Chinese ^G^γ^+^(^A^γδβ)^0^-thalassemia. However, it is difficult to distinguish the two by relying solely on hematological and HbF levels; accurate diagnosis relies on genetic testing.

## Conclusion

This is the first report to compare hematological parameters of the two common types of β-globin gene cluster deletion thalassemia in the high-incidence areas of β-thalassemia in the southern China. The precise molecular diagnosis and identification of rare variant of thalassemia can provide a better basis for clinical diagnosis and genetic counseling.

## Data Availability

The datasets analyzed during the current study are available from the corresponding author on a reasonable request.
